# Applying digital technologies for remote care in the real life context: A 3-year experimentation with postoperative lung cancer patients

**DOI:** 10.1097/MD.0000000000048750

**Published:** 2026-05-22

**Authors:** Dongfang Tang, Jing Wang, Wentao Fu, Fuzhi Yang, Nianping Mo, Saitian Li, Yuxu Niu, Yingting Wu, Huibiao Zhang, Xiaoyong Shen, Wen Gao

**Affiliations:** a Department of Thoracic Surgery, Shanghai Huadong Hospital, Fudan University School of Medicine, Shanghai City, China.

**Keywords:** digital technology, lung cancer, pulmonary function, remote care

## Abstract

Surgery remains the main curative treatment for lung cancer patients. However, operation has resulted in some-degree reduction in postoperative pulmonary function. The decreased pulmonary function could be rehabilitated to the preoperative level through restoring exercise, which is related to the ways and exercise time of rehabilitating. The Dignio platform is 1 digital telemedicine system and has been used maturely in Norway, however, it has not yet been widely promoted in China. In the present study, we adopted the telemedicine system, according to the formulated rehabilitation exercise program, to design a good way to recover pulmonary function for postoperative lung cancer patients, promoting early and quick recovery, and reducing the long-term postoperative complications and outpatient visits. This retrospective controlled study enrolled 250 postoperative lung cancer patients using a remote digital care platform and 250 matched controls receiving standard instructions. In the postoperative 1-week follow-up, we found that the forced expiratory volume in 1 second (FEV1), forced vital capacity (FVC), forced expiratory volume in 6 seconds, FVC25–75, peak expiratory flow had an obvious upward trend, although there were no significant differences in the measurements on postoperative day 7 compared with day 1. From the long-term data at 1 month/3 months/6 months post-operation, we found that the recovery was better to varying degrees in the experiment group using the remote care digital platform, compared with that of the control, especially the FEV1 at 3 (*P* = .065) and 6 months post-operation (*P* = .037), and forced Expiratory Flow between 25% and 75% of forced vital capacity at 3 months post-operation (*P* = .084). However, most pulmonary values, such as the FEV1/FVC, FVC and peak expiratory flow have no significant difference (all *P* >** **.05) in the recovery at 1 month post-operation between the experimental group and control, the second time at 3 months and the third time at 6 months after surgery. Especially in the experimental group, 2 patients with sudden pneumothorax were timely detected after discharge through the Digino platform, the patient returned to the hospital in time, and both patients were out of danger ultimately. The Digino remote care indeed has some benefit in the pulmonary function recovery in the long-term follow up, and reduces postoperative complications obviously.

## 1. Introduction

Lung resection remains the primary curative treatment for early-stage lung cancer,^[1]^ yet postoperative pulmonary function typically decreases by 10% to 40% due to lung tissue loss and altered chest wall mechanics.^[2-5]^ Although respiratory muscle strength may gradually recover after surgery, the early decline often persists longer than expected in clinical practice, even with minimally invasive techniques such as video-assisted thoracoscopic surgery.^[6-15]^ Inadequate recovery of pulmonary function can lead to long-term complications, including chronic cough, chest tightness, reduced exercise capacity, and increased airway reactivity.^[16-18]^ Evidence suggests that structured pulmonary rehabilitation can help restore function toward preoperative levels, with outcomes influenced by the type and duration of exercise.^[19-22]^

Digital health technologies have rapidly expanded and are now integral to modern healthcare systems.^[23,24]^ The COVID-19 pandemic further accelerated the adoption of remote care, particularly for chronic disease management and postoperative follow-up.^[25]^ Although digital rehabilitation platforms have been widely implemented in several countries, their use in China remains limited. Telemedicine-assisted rehabilitation may provide continuous monitoring of postoperative lung cancer patients, offer timely detection of complications such as pneumothorax or arrhythmia, and potentially improve adherence to rehabilitation programs.

Therefore, the present study aimed to evaluate whether a digitally enabled remote care system could enhance postoperative pulmonary function recovery, reduce complications, and decrease healthcare utilization among patients after lung resection.

## 2. Materials and methods

### 2.1. Dignio platform and APP

The Dignio prevent software platform has been developed in collaboration with patients and clinicians to guarantee safety and quality. From the patients, the App “gives patients” access to their data and a direct line of communication with healthcare professionals. Because the system is cloud-based, it provides patients with a large degree of independence.

The platform integrates data from a wide variety of home-based care and treatment methods, such as the medical measurements, questionnaires, medication, and rehabilitation – and gives healthcare professionals easy access to all the information they need. It guides patients to a healthier lifestyle and prevents hospitalization through early detection and intervention.

### 2.2. Wearable devices

Spirometry was bought from MIR (Spirobank), oximeter was bought from BLT (M70C), sphygmomanometer was bought from Yuwell (YE650A), ear thermometer was bought from MedLinket (IRT01B), electrocardiograph was bought from Beijing Haismin Medical Technology Co., Ltd (HM5100B).

These devices were associated with the Digino application (APP) in the mobile phone through Bluetooth, after the patient measures their vital signs, the results would be automatically uploaded to the platform through the APP and given symptomatic treatment or continuous follow-up according to the results.

**Figure 1.**
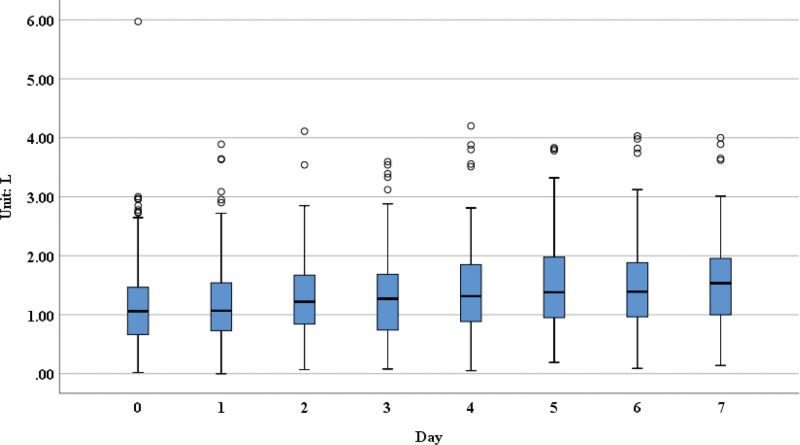
The FEV1 measurements over 7 days post-operation. FEV1 = forced expiratory volume in 1 second.

**Figure 2.**
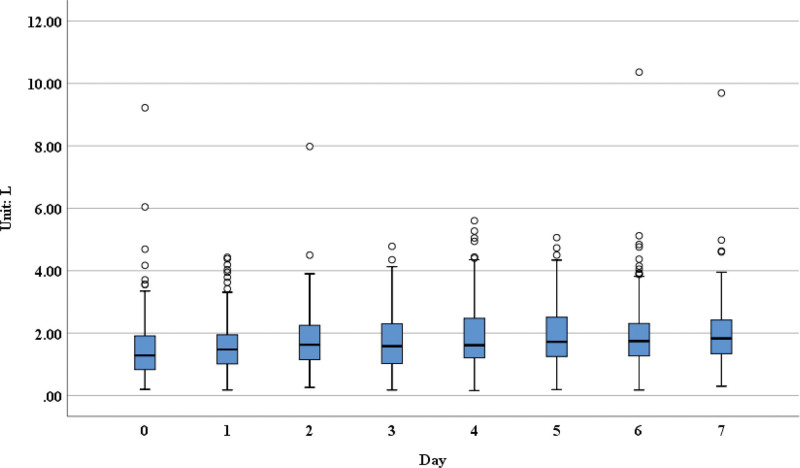
The FVC measurements over 7 days post-operation. FVC = forced vital capacity.

**Figure 3.**
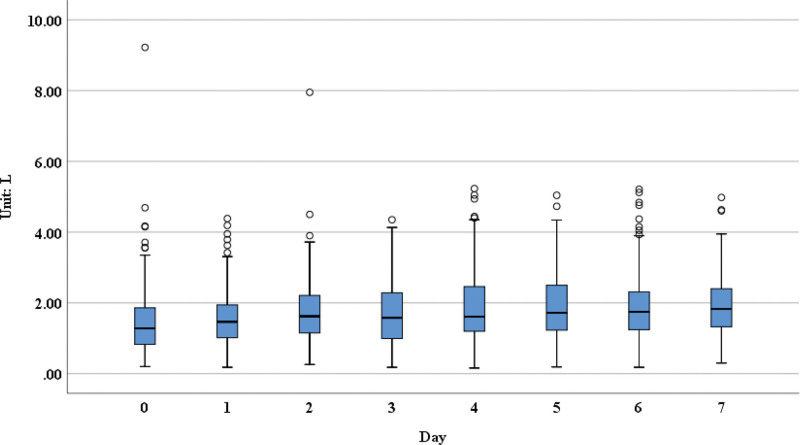
The FEV6 measurements over 7 days post-operation. FEV6 =forced expiratory volume in 6 seconds.

**Figure 4.**
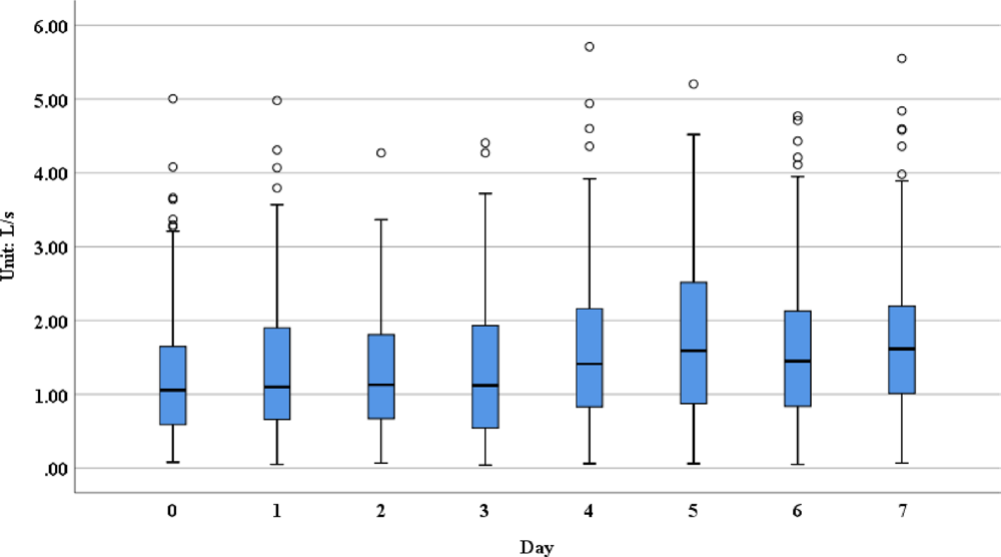
The FEF 25–75 measurements over 7 days post-operation. FEF 25–75 = forced expiratory flow from 25% to 75% of forced vital capacity.

### 2.3. Patient enrollment

This study was approved by the Ethics Committee of Shanghai Huadong Hospital (2023-SHHDH-kf134). There were 250 lung cancer patients enrolled in the present study from Mar 2020 to Oct 2022 at the Department of Thoracic Surgery in Huadong Hospital affiliated to Fudan University.

All patients were diagnosed with pulmonary mass lesions, and preoperative pulmonary function were normal. Patients who underwent thoracoscopic lobectomy or segmental or wedge resection successfully and recovered well after surgery.

All enrolled patients were instructed by personnel before discharge to download the APP for the use of wearable devices, as well as the exercise program for pulmonary function and precautions at home.

The control group enrolled 250 patients at the Department of Thoracic Surgery in Huadong Hospital affiliated to Fudan University, and all patients were also diagnosed with pulmonary mass lesions, and all patients underwent thoracoscopic lobectomy or segmental or wedge resection successfully and recovered well after surgery.

The signed informed consent was obtained from all patients and the clinical research ethics committee of Huadong Hospital affiliated to Fudan University approved the present study, all methods were carried out under relevant guidelines and regulations.

Unsimilar to the experimental group, the control was not suitable for the Digino platform and wearable devices, and they were also instructed on pulmonary function exercise programs and precautions after discharge (Table 1).

**Table 1:** Pulmonary function Rehabilitating program.

Time to discharge	Pulmonary training	Aerobic exercise
First week	15-25 min every morning and evening. Note: Keep breathing training but do not inhale deeply just after discharge, and deep breathing may lead to irritate cough or incision dehiscence	None
2 wk to 1 mo	15–25 min every morning and evening	2–3 times a week, each time lasting 10–20 min, please avoid excessive exercise may induce anaerobic exercise, and stop when you feel dizziness, palpitations, chest pain and other discomfort symptoms
Pulmonary function was measured a month after discharge		
1–3 mo	Once every morning and evening, keep exercising for 15–25 min each time	3–4 times per week, lasting about 30 min every time
Pulmonary function was measured 3 mo after discharge		
3–6 mo	Once every morning and evening, keep exercising for 15–25 min each time	3–4 times per week, lasting about 30 min every time

### 2.4. Monitoring content

Spirometry monitored the recovery of pulmonary function after rehabilitation exercise. oximeter monitored the changes of oxygen saturation every day, observed whether the patient was in an emergency of hypoxia or not, and warned the patients to visit hospital timely if necessary. Sphygmomanometer was used to observe the changes of blood pressure, and abnormal patients should be treated in time. Ear thermometer was to observe the change of patient ‘s body temperature; the electrocardiograph was to observe whether the patient had arrhythmia after operation. If the discharged patient was with emergency arrhythmia, and timely hospitalization was required.

### 2.5. Follow-up plan at home

The experimental group would use wearable devices and telemedicine for 1 to 2 weeks after discharge and monitor the above contents every day. They should keep rehabilitating according to the program. The wearable devices needed to be sent back 1 to 2 weeks later; the patients should go to examine the pulmonary function at 1 month, 3 months and 6 months after the operation and observe the recovery of pulmonary function.

The control did not use the telemedicine system and kept exercising according to the rehabilitating program. They also went to the hospital to examine the pulmonary function at 1 month, 3 months and 6 months after the operation.

### 2.6. First aid countermeasures

Once the Digino platform showed abnormal results from patients, such as hypoxemia, arrhythmia or other, the followed-up nurses would remind patients or their families to go to the hospital for treatment urgently.

### 2.7. Statistical analysis

Continuous variables were described in the form of mean ± standard deviation (SD) and categorical variables were described in the form of frequency (n) and percentage (%). Inter-group comparisons of continuous variables were performed using independent t test. The data analysis was conducted by SPSS 25.0 (Chicago) software, and the level of significance was set α = 0.05 at 2-tail.

## 3. Results

### 3.1. Established account number, patients’ information confidentiality and questionnaire

A new account could be established through inputting the patients’ mobile phone numbers on the Dignio platform, and the personnel input other patients’ information, such as age, gender, surgery, address and others, which could not be changed once established unless a new account was rebuilt. The information was only visible to managers of the platform and not to others.

After downloading the APP, the patient could enter the APP next time automatically after setting the password on the mobile phone. After the APP and wearable device were connected for the first time through Bluetooth, there was no need to repeat the connection later, the wearable device could be used directly, and the results were uploaded.

In addition to allowing the enrolled patients to use wearable devices, we also set up some questionnaires (Supplementary Questionnaire), which are different according to the recovery status, mainly for the patients’ complications. Generally, 1 month, 3 months and 6 months after operation were turning points, and patients could automatically jump into the next questionnaire after completing the previous 1.

The platform has a dedicated nurse who handles the data uploaded by the patients and communicates with them. If there were urgent values, the nurse would contact the family to confirm whether the result was wrong or the patient had an emergency, if confirmed later, she would contact the doctor to give emergency symptomatic treatment.

### 3.2. Basic characteristics of patients

The characteristics of the experimental group and control group are in Table 2.

**Table 2:** The basic characteristics of 2 groups.

	Experimental group	Control group	*P*
Age			
	53.11 ± 16.09	58.24 ± 12.47	>.05
Gender			
Male	111	81	
Female	139	169	.0076
Surgery			
Lobectomy	128	113	
Non-Lobectomy	122	137	.210
Complication			
Yes	2	1	
No	248	249	>.05

Except for the gender (proportion of male: 44.44% vs 32.18%, *P* = .0076), It could be found that there were no differences in age (53.11 ± 16.09 vs 58.24 ± 12.47, *P* > .05), and surgery (*P* > .05) between the experimental group and control. In terms of complications, there was no difference too (*P *> .05). There were 2 patients with sudden pneumothorax after discharge in the experimental group and none in the control.

### 3.3. Short-term pulmonary function follow-up results

In the experimental group, pulmonary function and vital signs were monitored for 1 week using wearable devices from the first day of discharge.

From our results, we could see that the forced expiratory volume in 1 second (FEV1; Fig. 1), forced vital capacity (FVC; Fig. 2), forced expiratory volume in 6 seconds (Fig. 3), FVC25–75 (Fig. 4), peak expiratory flow (PEF; Fig. S1) had obvious upward trend, indicating that patients’ pulmonary function was recovering gradually after lung surgery through the pulmonary rehabilitation guidance and the use of digital platform, although there were no significant differences in the measurements on postoperative day 7 compared with day 1.

The temperature (Fig. S2) had an upward trend at the beginning, and gradually came back to normal later; we inferred that it was the absorption heat induced by the absorption of residual pleural effusion after the drainage tube removal in pneumonectomy patients; the temperature returned to normal after the effusion was absorbed completed. The oxygen saturation concentration has been maintained normal (Fig. S3), indicating that most patients continued to have normal oxygen saturation after surgery, and there were no complications such as pulmonary embolism and so on.

### 3.4. Long-term pulmonary function follow-up results

From the long-term data at 1 month/3 months/6 months post-operation, we found that the recovery was better to varying degrees in the experiment group using the remote care digital platform, compared with that of the control group, especially the FEV1 at 3 (*P* = .065, Table 3) and 6 months post-operation (*P* = .037, Table 2), and forced Expiratory Flow between 25% and 75% of forced vital capacity at 3 months post-operation (*P* = .084, Table S1). Whereas, the blood pressure, oxygen saturation concentration and temperature remained normal, did not have substantial rise, or fall.

**Table 3:** Postoperative FEV1 measurements in the experimental and control group.

FEV1	Experimental group				Control group				*P* *
	n	Mean ± SD	Min	Max	n	Mean ± SD	Min	Max	
1 mo post-operation	11	2.23 ± 0.75	1.07	3.65	75	2.09 ± 0.58	0.93	3.50	.474
3 mo post-operation	8	2.59 ± 0.73	1.62	3.67	25	2.11 ± 0.58	1.13	3.21	.065
6 mo post-operation	5	2.26 ± 0.36	1.90	2.76	4	1.66 ± 0.33	1.43	2.14	.037
9 mo post-operation	1	1.81	1.81	1.81	0	–	–	–	–

FEV1 = forced expiratory volume in 1 second, SD = standard deviation.

* Two indepedent *t*-test.

However, most pulmonary values, such as the FVC (Table 4), FEV1/FVC (Table 5), and PEF (Table S2) have no significant difference (all *P* > .05) in the recovery at 1 month post-operation between the experimental group and control, the second time at 3 months and the third time at 6 months after surgery. The results did not seem to be as we initially envisioned. As a fact, the results revealed that any wearable device did not interfere with the physiological recovery.

**Table 4:** Postoperative FVC measurements in the experimental and control group.

FVC	Experimental group				Control group				*P* *
	n	Mean ± SD	Min	Max	n	Mean ± SD	Min	Max	
1 mo post-operation	11	2.88 ± 0.72	1.97	4.41	75	2.54 ± 0.73	1.09	4.07	.152
3 mo post-operation	8	3.07 ± 0.89	2.10	4.65	25	2.67 ± 0.74	1.35	4.36	.214
6 mo post-operation	5	2.94 ± 0.77	2.26	4.02	4	2.15 ± 0.53	1.55	2.84	.126
9 mo post-operation	1	1.98	1.98	1.98	0	–	–	–	–

FVC = forced vital capacity, SD = standard deviation.

* Two independent *t*-test.

**Table 5:** Postoperative FEV1/FVC measurements in the experimental and control group.

FEV1/FVC	Experimental group				Control group				*P* *
	n	Mean ± SD	Min	Max	n	Mean ± SD	Min	Max	
1 mo post-operation	11	77.47 ± 16.77	34.23	91.81	75	83.29 ± 9.10	65.50	100	.086
3 mo post-operation	8	84.59 ± 5.65	77.05	91.86	25	79.67 ± 8.57	66.77	93.19	.142
6 mo post-operation	5	79.01 ± 7.72	68.77	85.62	4	78.38 ± 11.27	67.53	94.20	.923
9 mo post-operation	1	91.48	91.48	91.48	0	–	–	–	–

FEV1/FVC = forced expiratory volume in 1 second/forced vital capacity, SD = standard deviation.

* Two independent *t*-test.

It was noteworthy that the wearable devices and remote monitoring platforms played a non-negligible effect. It could effectively guide patients to carry out the exercise, reduce the incidence of postoperative complications significantly, such as intractable cough and other complications that seriously affect the quality of life, and improve the patient’ sense of postoperative comfort.

Although more high-intensity training may be required to restore the preoperative pulmonary function, however, from our results, we found that the recovery was better in the long term relying on postoperative guidance and digital remote care platform, considering the surgical trauma, we recommend that the patients started intensive aerobic exercise 1 month after surgery generally.

### 3.5. Management of emergent complications after discharge

In the experimental group, we found 2 patients with sudden pneumothorax after discharge through the Digino platform, mainly judged by abnormal pulmonary function, oxygen saturation <95%, and their symptoms feedback by the patients through the Digino platform. Then we contacted the doctor in charge, the patient returned to the hospital in time, and both patients were out of danger through emergency treatment.

## 4. Discussion

In the present study, we found that the long-term pulmonary recovery was better to some degree in the experiment group after the application of remote digital platform, compared with the control, such as the FEV1 and forced Expiratory Flow between 25% and 75% of forced vital capacity. Whereas, most pulmonary function values, such as the FEV1/FVC, FVC and PEF have no significant difference. It’s not difficult to understand the results based on the medical knowledge, the recovery of pulmonary function was a physiological process, and external intervention could not accelerate its rehabilitation. It may be attributed to our short follow-up time, and some positive results may be obtained if follow-up was longer. However, the use of digital remote care does reduce postoperative complications, such as long-term persistent cough, chest tightness, suffocation and others, therefore it is worthy to affirm that it has improved the quality of postoperative patients’ lives.

Although we have achieved some meaningful results, we have encountered a lot of difficulties in adapting the system and applying the system to patients, the main problems we encountered during the study were as follows, first, system compatibility was a big challenge, particularly at the beginning. Patients had all kinds of problems when trying to download and install the APP on their mobile phones, such as some phone models could not download and install this App, and others could not connect to wearable devices through Bluetooth. In addition, the accuracy of data acquisition was also a challenge, some patients had difficulty using the medical devices to measure vital signs correctly. Another problem was that enrolling patients was not a straightforward process; some patients might not be properly informed about the importance of postoperative rehabilitation. Patients might not trust when they first encountered new technologies. Several patients tended to be less motivated to install and learn a new mobile application. There was also a lack of engagement in the hospital, the project was carried out through the thoracic surgery department only, resulting in limited human resources available for providing digital remote care services. This may not provide high-quality care experience for patients in the long run. Therefore, poor patient compliance has been the biggest challenge during the project. The remote care solution seemed cost-efficient in the sense that the patients would reduce the need to revisit the hospitals after being discharged, however, the present results were achieved based on nearly twenty personnel in the hospitals who worked close to voluntary labor. Therefore, the real costs need to be further calculated when the sample size is larger to evaluate if there is a significant economic benefit.

The efficacy of remote care digital platforms depended on their using design, when the digital platform was used in a specific patient population, their impact on patients’ outcome was higher. It was also necessary to make the feedback data collected in a more meaningful way for patients. The digital platform helped patients gain autonomy and control over their current health problems. The data was stored in a central database, the clinical information could be available by multidisciplinary team in real-time. So, it allowed the doctors to monitor the disease evolution in an outpatient setting. Furthermore, patients receiving feedback from the system or health professionals after submitting the questionnaire revealed higher satisfaction with the team’s follow-up. It was gratifying that 2 patients with delayed pneumothorax and 1 patient with pericardial effusion were found in time according to the abnormal monitoring values received during the present study, and these 3 patients were sent to the hospital in time to save their lives and recovered, this was considered to be the greatest advantage for the digital remote care system during the follow-up of patients after cardiothoracic surgery.

From our experience, the digital platform could facilitate communication between patients and health care providers in several ways: Patients engaged more: the digital platform enabled patients to actively participate in their care through self-assessing their symptoms and functional status. More completed patients’ data: the digital platform provided health care providers with additional patient-generated data and help providers to make more accurate assessments. Timely assessments: the digital platform allowed patients to receive reminders to complete assessments at their convenience. Remote monitoring: the digital platform could be used to monitor patients remotely and submitted electronically. Real-time feedback: this could help health care providers adjust their treatment plans as soon as possible. The study encountered several practical challenges. System compatibility issues affected some patients’ ability to install or connect wearable devices, and compliance varied among elderly patients unfamiliar with digital tools. Data acquisition accuracy was occasionally limited by user-related factors. Moreover, although remote care appeared cost-efficient, substantial human resources were required during the study, suggesting that the true economic impact requires further evaluation in larger cohorts.

After a period of use, the patient has found the advantages of this remote care system, most patients who were exposed to the digital platform found it was easy and useful, would like to recommend it to other patients and continue to use it. However, a small population still showed reluctance to accept the remote submission of the clinical questionnaires instead of the traditional. In all, the digital platform could facilitate communication between patients and health care providers through providing timely patient’s data, enabling more efficient assessments.

## Author contributions

**Conceptualization:** Dongfang Tang, Jing Wang, Wentao Fu, Nianping Mo, Saitian Li, Yuxu Niu, Yingting Wu, Wen Gao.

**Data curation:** Dongfang Tang, Jing Wang, Wentao Fu, Fuzhi Yang, Nianping Mo, Saitian Li, Yuxu Niu, Yingting Wu, Xiaoyong Shen, Wen Gao.

**Formal analysis:** Dongfang Tang, Jing Wang, Wentao Fu, Fuzhi Yang, Nianping Mo, Yuxu Niu, Yingting Wu, Huibiao Zhang, Xiaoyong Shen, Wen Gao.

**Funding acquisition:** Dongfang Tang, Nianping Mo, Yingting Wu, Huibiao Zhang, Xiaoyong Shen, Wen Gao.

**Investigation:** Nianping Mo, Huibiao Zhang, Wen Gao.

**Writing – original draft:** Dongfang Tang, Jing Wang, Wentao Fu, Fuzhi Yang, Saitian Li, Yuxu Niu, Huibiao Zhang, Xiaoyong Shen, Wen Gao.

**Writing – review & editing:** Dongfang Tang, Jing Wang, Wentao Fu, Fuzhi Yang, Saitian Li, Yuxu Niu, Huibiao Zhang, Xiaoyong Shen, Wen Gao.












