# Supplementary material for: Applying digital technologies for remote care in the real life context: A 3-year experimentation with postoperative lung cancer patients
Source: Medicine (Baltimore). 2026 May 22;105(21):e48750. doi: 10.1097/MD.0000000000048750 (PMC13200953; doi:10.1097/MD.0000000000048750)
Supplement: Supplementary file 1 [file medi-105-e48750-s001.docx]

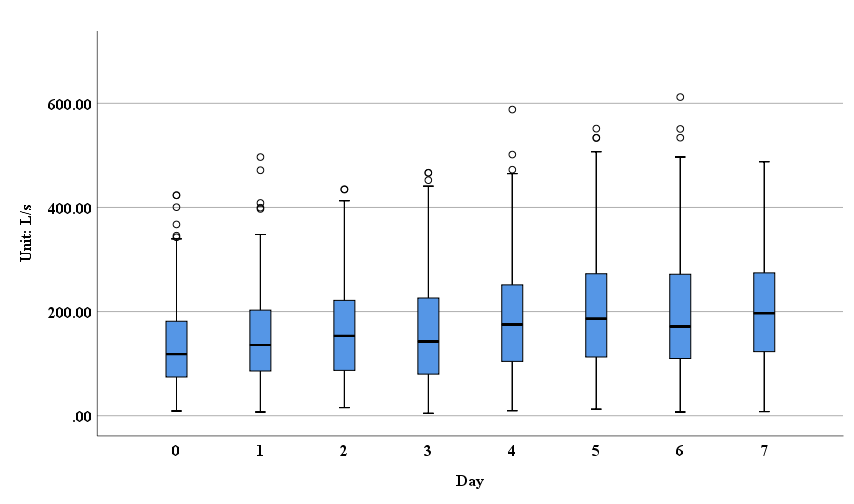
 **Supplemental Figure 1** The PEF measurements over 7 days post-operation.

**Note:** FEF, Forced expiratory flow.
