# Supplementary material for: Applying digital technologies for remote care in the real life context: A 3-year experimentation with postoperative lung cancer patients
Source: Medicine (Baltimore). 2026 May 22;105(21):e48750. doi: 10.1097/MD.0000000000048750 (PMC13200953; doi:10.1097/MD.0000000000048750)
Supplement: Supplementary file 2 [file medi-105-e48750-s002.docx]

**Supplemental Table 1** Postoperative FEF 25-75 measurements in the experimental and control group

| **FEF 25-75** | Experimental group | | | |  | Control group | | | | *P*** |
| --- | --- | --- | --- | --- | --- | --- | --- | --- | --- | --- |
|  | n | Mean ± SD | Min | Max |  | n | Mean ± SD | Min | Max |  |
| 1m post-op^*^ | 11 | 2.24±0.94 | 0.74 | 3.43 |  | 75 | 2.15±0.99 | 0.42 | 4.79 | 0.778 |
| 3m post-op^*^ | 8 | 2.71±0.91 | 1.26 | 4.08 |  | 25 | 2.08±0.85 | 0.67 | 3.56 | 0.084 |
| 6m post-op^*^ | 5 | 1.98±0.39 | 1.64 | 2.58 |  | 4 | 1.46±0.58 | 0.76 | 2.08 | 0.151 |
| 9m post-op^*^ | 1 | 2.35 | 2.35 | 2.35 |  | 0 | - | - | - | - |

Note, *: post-operation, **: two independent t-test.

FEF25-75, Forced expiratory flow from 25-75% of FVC.
