# Supplementary figures and images for: Applying digital technologies for remote care in the real life context: A 3-year experimentation with postoperative lung cancer patients
Source: Medicine (Baltimore). 2026 May 22;105(21):e48750. doi: 10.1097/MD.0000000000048750 (PMC13200953; doi:10.1097/MD.0000000000048750)

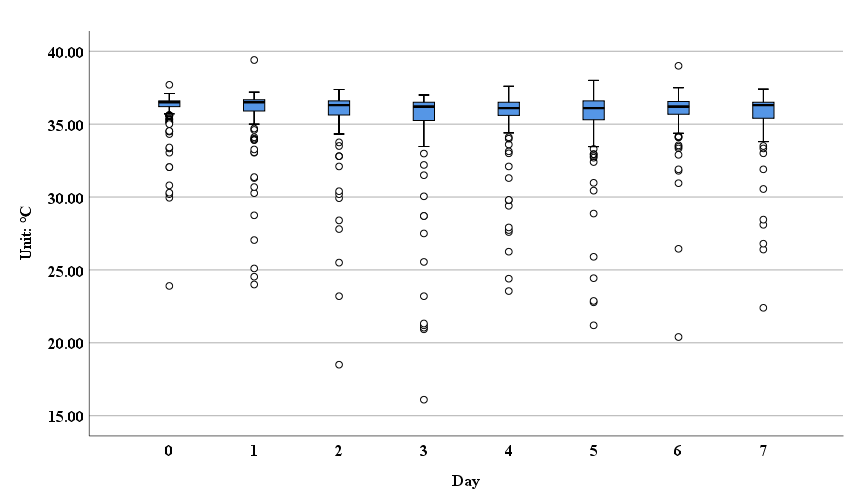


**Supplemental Figure 2** The body temperature measurements over 7 days post-operation.

Supplement: Supplementary file 3 [file medi-105-e48750-s003.docx]

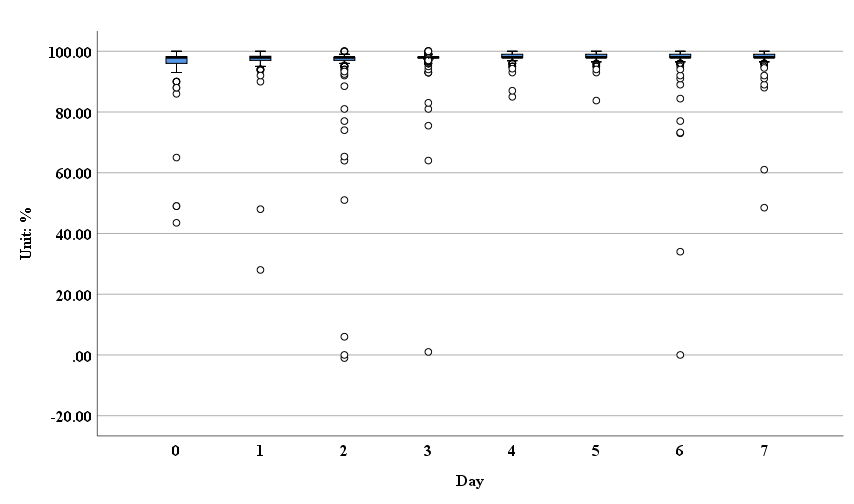


**Supplemental Figure 3** The oxygen Saturation measurements over 7 days post-operation.

Supplement: Supplementary file 4 [file medi-105-e48750-s004.docx]
