# Supplementary material for: Applying digital technologies for remote care in the real life context: A 3-year experimentation with postoperative lung cancer patients
Source: Medicine (Baltimore). 2026 May 22;105(21):e48750. doi: 10.1097/MD.0000000000048750 (PMC13200953; doi:10.1097/MD.0000000000048750)
Supplement: Supplementary file 5 [file medi-105-e48750-s005.docx]

**Supplemental Table 2** Postoperative PEF measurements in the experimental and control group

| **PEF** | Experimental group | | | |  | Control group | | | | *P*** |
| --- | --- | --- | --- | --- | --- | --- | --- | --- | --- | --- |
|  | n | Mean ± SD | Min | Max |  | n | Mean ± SD | Min | Max |  |
| 1m post-op^*^ | 11 | 5.42±2.04 | 1.44 | 7.75 |  | 75 | 5.34±1.78 | 1.32 | 9.32 | 0.892 |
| 3m post-op^*^ | 8 | 6.11±2.42 | 4.26 | 10.72 |  | 25 | 5.10±1.52 | 2.23 | 8.94 | 0.169 |
| 6m post-op^*^ | 5 | 6.31±0.99 | 5.34 | 7.58 |  | 4 | 4.23±1.52 | 2.48 | 6.66 | 0.058 |
| 9m post-op^*^ | 1 | 4.55 | 4.55 | 4.55 |  | 0 | - | - | - | - |

Note, *: post-operation, **: two independent t-test.

FEF, Forced expiratory flow.
