# Supplementary material for: Applying digital technologies for remote care in the real life context: A 3-year experimentation with postoperative lung cancer patients
Source: Medicine (Baltimore). 2026 May 22;105(21):e48750. doi: 10.1097/MD.0000000000048750 (PMC13200953; doi:10.1097/MD.0000000000048750)
Supplement: Supplementary file 6 [file medi-105-e48750-s006.docx]

**Quality of Life Questionnaire for patients after video-assisted thoracoscopic surgery (VATS)**

**Periodic questionnaire：**

Note: We could get the corresponding contents of patients in the control group by telephone or WeChat.

**Introduction**

You are going to fill in this questionnaire several times via this platform, such as 7 days, 1 month, 3 months, 6 months, 9 months and 1 year after the surgery so that our doctors could have better management of your rehabilitation.

*All the events asked below should take place since the last time they fill in the questionnaire.*

1. **Questions about cardiopulmonary function**

**Do you have a cough or do you cough up phlegm?**

1.Never.

2.Sometimes, and it doesn’t influence life and work.

3.Often, and it influences life and work to some extent.

4.Often, and it influences life and work greatly.

**Have you** **developed hemoptysis or sputum with blood?**

1.Never.

2.Sometimes I cough up sputum with blood.

3.I often cough up sputum with blood.

4.I have hemoptysis.

**Do you have symptoms of chest tightness or shortness of breath?**

1.Never.

2.Yes, but it makes no influence on daily activities.

3.Yes, but it influences daily activities slightly.

4.Yes, I immediately feel uncomfortable once moving. Or I have symptoms of chest tightness or shortness of breath even when I am taking a rest. Or I feel the symptoms is becoming more and more serious.

**Do you have chest pain?**

1.Never.

2.Yes, but it makes no influence on daily activities.

3.Yes, but it influences daily activities slightly.

4.Yes, I immediately feel uncomfortable once I move. Or I have symptoms of chest pain even when I am taking a rest. Or I feel the symptoms is becoming more and more serious.

**B. Questions about performance status**

**Could you carry out daily activities normally?**

1. I could normally carry out daily activities, and general daily activities do not cause discomfort. /I’m 80 years old or older, I could take care of myself independently.

2.I have a little difficulty in carrying out daily activities.

3.Obviously I have difficulty in carrying out daily activities. And I couldn’t walk more than 1 kilometer in an average speed or I couldn’t ascend one  story of the building.

4.I feel discomfortable when I take light activities, or I need to stay in bed for more than half of the daytime.

**C. Questions about self-care ability.**

**Do you need other people to help you with daily self-care activities such as brushing teeth, washing face, dressing up, eating meals, etc.**

1.No.

2.Yes, I depend partly on others.

3.Yes, I depend completely on others.

**D. Questions about pain or discomfort**

**Please score according to the extent of pain. (Reference: 0 painless 1-3 mild pain 4-6 moderate pain 7-10 severe pain)**

| 0 | 1 | 2 | 3 | 4 | 5 | 6 | 7 | 8 | 9 | 10 |
| --- | --- | --- | --- | --- | --- | --- | --- | --- | --- | --- |

**Do you need medication to relieve the pain?**

1.No.

2.I take non-steroidal anti-inflammatory drugs(NSAIDS), such as Fenbide, celecoxib, etocoxib, etc.

3.I take weak opioids, such as tramadol hydrochloride sustained-release tablets, oxycodone, etc.

4.I take strong opioids, such as morphine, Oxycodone sustained-release tablets, fentanyl patches.

5.I take analgesics, but I do not know which classification it belongs to, List the name of analgesics you take, ______________.

**E. The effect of disease on mental state**

**Do you have the tendency of anxiety or depression?**

1.No.

2.I have mild anxiety or depression, but it doesn’t affect daily life and sleep.

3.I have moderate anxiety or depression, and it makes adverse effect on daily life and sleep.

4.I have severe anxiety or depression, and most of the time I couldn’t conduct daily work or study.

**F. Adverse event, AE**

**Have you visited the outpatient or emergency department of thoracic surgery again due to discomfort?**

1.No.

2.Yes, list when and why you saw the doctor of thoracic surgery department because of discomfort since last time you filled in this type of questionnaire

**Have you been hospitalized again in the thoracic surgery department due to discomfort？**

1.No.

2.Yes, list when and why you were hospitalized in the thoracic surgery department due to discomfort since last time you filled in this type of questionnaire, and write down the approximate length of hospitalization each time. Mark hospitalization in our hospital__________________

**Have you been hospitalized in other departments due to discomfort?**

1.No.

2.Yes, Why? ________________

**Have you ever had atrial fibrillation or other arrhythmias? (confirmed by ECG)**

1.No.

2.Yes, What type? _____________

**Any event of bleeding？**

1.No.

2.Yes, teeth, gums, nasal bleeding, Subcutaneous bleeding, hemorrhoids bleeding.

3.Yes, hematuria, hemoptysis.

4.Yes, gastrointestinal bleeding([melena](http://dict.youdao.com/w/melena/#keyfrom=E2Ctranslation)), strokes and cerebral hemorrhage.

5.Death due to bleeding.

**Whether the following events occurred during the observation:**

(1) Death

(2). Hospitalization or prolonged hospitalization

(3) Persistent or serious disability/dysfunction

(4) Life-threatening

(5) Congenital anomaly/birth defect

(6) Other, please specify

**Concomitant medication:**

Whether the medication has changed, and it is necessary to consider the medication at admission.

For each drug: set the date, when to stop, dose reduction, dose increase.

**Exercise, diet and drugs:**

1. Fully following the doctor 's advice.

2. Basically follow the doctor 's advice, and there are no more than three records of failure to follow the doctor' s advice during the observation.

3. Poor compliance with medical orders, with more than three records of non-compliance during the observation.

**G. Comments on service**

**Whether problems of disease or pulmonary function rehabilitation could be solved in time via this platform?**

1.It does not help.

2.Some of them.

3.Most of them.

4.All of them.

**Do you feel more connected with the doctor through this platform?**

1.No.

2.Yes. Any suggestion？___________________________________________

**Daily questionnaire**

**You should fill in this questionnaire every day until 7 days after discharge.**

**Have you completed your daily exercise？**

1.No. Why? ____________

2.Yes, most of it.

3.Yes, I complete all.

**Have you taken drugs as prescribed by doctors which is necessary for your diseases?**

1.Never.

2.Sometimes, I forget.

3.I take medicine strictly according to the doctors’ advice.

**Diet**

1.Poor appetite.

2.Normal appetite, but I pay little attention to nutrition.

3.Normal appetite, nutritious diet. I pay attention to the combination of nutritional elements.

**Whether you cough or cough up phlegm or not today?**

1.No

2.Yes

**Whether you develop hemoptysis or sputum with blood or not today?**

1.No

2.Yes

**Whether you have symptoms of chest tightness or shortness of breath or not today?**

1.No

2.Yes

**Whether you have chest pain or not today?**

1.No

2.Yes

**Whether you have a fever or not today?**

1.No

2.Yes

**Whether you have any other discomfort or not today? If the answer is yes, tell us more about it.**

1.No

2. Yes______________
